# Radiation-Grafted Anion-Exchange Membrane for Fuel Cell and Electrolyzer Applications: A Mini Review

**DOI:** 10.3390/membranes11060397

**Published:** 2021-05-27

**Authors:** Kean Long Lim, Chun Yik Wong, Wai Yin Wong, Kee Shyuan Loh, Sarala Selambakkannu, Nor Azillah Fatimah Othman, Hsiharng Yang

**Affiliations:** 1 Fuel Cell Institute, Universiti Kebangsaan Malaysia, Bangi 43600, Malaysia; nickwcy72@gmail.com (C.Y.W.); waiyin.wong@ukm.edu.my (W.Y.W.); ksloh@ukm.edu.my (K.S.L.); 2 Radiation Processing Technology Division, Malaysia Nuclear Agency, Kajang 43000, Malaysia; sarala@nuclearmalaysia.gov.my (S.S.); azillah@nm.gov.my (N.A.F.O.); 3 Graduate Institute of Precision Engineering and Innovation and Development Center of Sustainable Agriculture (IDCSA), National Chung Hsing University, 145 Xingda Road, South District, Taichung City 402, Taiwan

**Keywords:** grafting, irradiation, green hydrogen, alkaline fuel cell, alkaline water electrolysis

## Abstract

This review discusses the roles of anion exchange membrane (AEM) as a solid-state electrolyte in fuel cell and electrolyzer applications. It highlights the advancement of existing fabrication methods and emphasizes the importance of radiation grafting methods in improving the properties of AEM. The development of AEM has been focused on the improvement of its physicochemical properties, including ionic conductivity, ion exchange capacity, water uptake, swelling ratio, etc., and its thermo-mechano-chemical stability in high-pH and high-temperature conditions. Generally, the AEM radiation grafting processes are considered green synthesis because they are usually performed at room temperature and practically eliminated the use of catalysts and toxic solvents, yet the final products are homogeneous and high quality. The radiation grafting technique is capable of modifying the hydrophilic and hydrophobic domains to control the ionic properties of membrane as well as its water uptake and swelling ratio without scarifying its mechanical properties. Researchers also showed that the chemical stability of AEMs can be improved by grafting spacers onto base polymers. The effects of irradiation dose and dose rate on the performance of AEM were discussed. The long-term stability of membrane in alkaline solutions remains the main challenge to commercial use.

## 1. Overview of Anion Exchange Membrane for Fuel Cell (AEMFC) and Electrolysis Cell (AEMEC) Applications

The alkaline fuel cell was among the first commercial hydrogen-oxygen fuel cells invented in the 20th century, and it was used in the NASA Apollo space mission program in the 1960s. The reverse reaction of this hydrogen-oxygen combination process is electrolysis, and the alkaline electrolysis cell is one of the reliable and mature water splitting technologies used to produce hydrogen on a large scale. Both technologies are viewed as a sustainable solution to bridge the renewable energy sources to end-users that use green hydrogen as the carbon-free energy carrier. While the proton exchange membrane (PEM) fuel cell has gained more popularity [1,2,3,4,5] than the alkaline fuel cell in low-temperature applications, alkaline electrolyzer remains widely deployed for large-scale hydrogen production from renewable energy. Both these alkaline technologies are relatively cost effective because non-precious metal electrocatalysts show favorable, stable and efficient performance towards oxygen reduction (for fuel cell) as well as evolution (for electrolyzer) reactions, which are attained in an alkaline environment. Nevertheless, both technologies use strong alkaline aqueous solution (i.e., KOH) as an electrolyte that unfortunately can react with carbon dioxide in the air to produce insoluble precipitates (i.e., K_2_CO_3_), leading to the blockage of catalyst active sites and porous separator as well as the reduction of hydroxyl ions in anolyte, which eventually reduce the overall performance of the devices. In addition, the alkaline fuel cell and electrolyzer with liquid electrolyte [6] are inherently troublesome to shut down/start up, prone to leaking, have a high concentration gradient, and require consistent and stable output conditions. Leveraging on the knowledge of proton exchange membrane (PEM), solid-state anion exchange membrane (AEM), which offers the similar advantages of alkaline liquid fuel cell/electrolyzer, has been proposed as the replacement for liquid electrolyte. With the recent announcement of the Green Deal by the European Union and gaining interest of green hydrogen production from water electrolysis, AEM electrolysis has been viewed as a potential technology that could reduce the hydrogen production cost significantly to the targeted price of USD 4/kg H_2_ [7,8]. 

Merle et al. [9] have classified the AEMs into three categories: heterogenous membranes, interpenetrating polymer networks and homogeneous membranes. Heterogenous membrane can be prepared by embedding anion exchange material in an inert compound (either hydroxide salt or inorganic nanoparticles), whereas interpenetrating polymer networks is an amalgamation of two polymers in the form of a network, where both polymers are either partially or fully crosslinked. Most AEMs are homogeneous membranes and typically can be prepared via direct polymerization and crosslinking or the chemical modification of polymers by irradiation, grafting or chemical reactions. Homogeneous anion exchange membrane is defined by a semi-permeable membrane that consists of positively charged cation head groups that are immobilized in the polymer backbone that allow for the creation of conduction channels for the mobile, negatively charged anions to transport through the membrane [10]. The immobilization of cation head groups also limits their reaction and contact with carbon dioxide to reduce and prevent the formation of carbonate precipitates [11]. The polymer backbones of AEM are usually based on polysulfone, poly(arylene ether), poly(phenylene), polystyrene, polypropylene, poly(ether sulfone), poly(phenylene oxide), polyolefin, poly(arylene piperidinium) and poly(biphenyl alkylene) [8,12]. In comparison to PEM, the performance of AEM is admittedly lower due to the higher ion mobility in H^+^ (4.76) compared to OH^−^ (2.69) [13]. In an anion exchange membrane fuel cell (AEMFC), oxygen is reduced at the cathode side and to produce hydroxide (OH^−^) ions and electrons, where hydroxide ions transport through the AEM towards the anode side and subsequently react with hydrogen to produce water, whereas the electrons travel through the external circuit to produce electricity current. Equations (1)–(3) describe the electrochemical reactions at the cathode side, anode side and overall reaction, respectively. 

Cathode:

(1) $$O_{2}+{2\;H}_{2}{O+4\;e}^{-}\to 4{\;\mathrm{OH}}^{-},$$

Anode:

(2) $$2{\;H}_{2}+4{\;\mathrm{OH}}^{-}\;\to \;4{\;H}_{2}O\;+4{\;e}^{-},$$

Overall:

(3) $$2{\;H}_{2}+O_{2}\;\to 2\;H_{2}O\;,$$

On the other hand, the anion exchange membrane electrolysis cell (AEMEC) uses electricity to split the water molecules into hydrogen gas and hydroxide ions at the cathode side, and the hydroxide ions transport through the membrane and eventually oxidize to produce oxygen gas and water, as shown in Equations (4)–(6). Ideally, water should be fed and consumed at the cathode, but many reported systems still feed hydroxide-ion-rich alkaline solutions to the cathode. 

Cathode:

(4) $$4\;H_{2}{O+4\;e}^{-}\to 2\;H_{2}+4{\;\mathrm{OH}}^{-},$$

Anode:

(5) $$4\;{\mathrm{OH}}^{-}\to O_{2}+{2H}_{2}O\;{+4\;e}^{-},$$

Overall:

(6) $$2\;H_{2}O\;\to 2{\;H}_{2}+O_{2},$$

While the efficiency of both electricity generation and water splitting depends highly on the performance of overall membrane electrode assembly (MEA) [14], which includes the catalyst, gas diffusion layers and electrolyte membrane, the mobility of OH^−^ in the membrane plays the most essential role in dictating the overall fuel cell/electrolysis cell performance. In addition to high anion conductivity, high thermo-mechanical and chemical stability properties of AEM are also highly sought after. Selection of cationic head groups, morphology design of membrane, product scalability, cost of raw materials and fabrication methods are factors to be considered and optimized to meet the commercial viability criteria [15]. 

## 2. Modification of Anion-Exchange Membrane 

In the past, a conventional process “polymerization-chloromethylation-amination” is usually employed to prepare AEMs [16]. This process is relatively mature and provides good performance for the membrane. However, the chloromethylation agent (chloromethyl ether) being used over that time had proven to be carcinogenic and hazardous [17]. Thereof, the alternative in the co-polymerization of vinylbenzyl chloride (VBC) with divinylbenzene (DVB), the grafting of VBC or vinylpyridine onto polymer, and the copolymerization of epoxy acrylates such as glycidyl methacrylate (GMA) have been reported as the halomethyl-substituted monomers to replace the usage of chloromethyl ether [18]. Aside from that, a variety of cationic functional groups have been proposed for AEM applications such as quaternary ammonium, imidazolium, pyridinium, guanidinium, phosphonium and sulfonium [19,20,21,22,23,24,25,26]. Quaternary ammonium ions have been traditionally used as the cation head group, but they are susceptible to chemical degradation via (i) Hofmann elimination, (ii) nucleophilic substitution and (iii) ylide formation [27]. One of the most important criteria in the selection of cation functional group is centered on its chemical stability under alkaline conditions. Hence, other nitrogen-containing cations (imidazolium, pyridinium and guanidinium) and nitrogen-free cations (phosphonium and sulfonium) head groups are also considered for AEM applications. Though a huge number of studies have been conducted in relation to AEMs, there are challenges in practical applications that remain to be solved, for instance, the low ionic conductivity of AEMs and low chemical stability of cationic functional groups as well as the polymer backbone in alkaline media [28,29,30]. Studies have shown that different fabrication methods even on the same polymer type could result in different properties. According to Maurya et al. [18], the final properties of the AEMs are intimately linked to the methods and materials used in fabricating an AEM. By integrating different polymer fabrication processes and altering the morphology of polymers, the performance and characteristics of AEM can be engineered. Generally, AEMs are prepared through three main steps, including the polymerization of halomethyl-substituted monomers, film casting and quaternization to create the cation head group on the polymeric film. The formation of the film is normally performed via solution casting or the sol-gel technique. Meanwhile, on the attempt to improve the thermo-mechanical, chemical stability, and ion conductivity properties of AEMs, various modification methods have been investigated by employing different polymers and reagents. Among the most commonly reported methods are direct copolymerization, plasma polymerization, the pore-filling method, and supported composite AEMs [18,31,32,33]. Copolymerization, which involves the use of halomethyl-substituted monomers as copolymer, which is required to serve as the site for quaternization in the subsequent process, is one of the crucial methods to create the anions’ transport sites on the membrane. For AEMs, the grafted co-polymerization technique, where the side chain grafts of the co-polymer are covalently attached to the polymer main chain, is more favorable compared to blocked copolymerization to increase the active sites for anion transport. Radiation-grafted copolymerization has emerged as the preferred technique, owing to its ease in controlling the degree of grafting, its ability to scale-up and the fact that it can minimize the residues left on the membrane during the process. This section will compare the different modification methods towards several properties of AEMs, including the ionic conductivity summarized in Table 1. Radiation-grafted AEMs will be highlighted based on their superior properties compared to other methods. 

### 2.1. AEM Prepared via Solution Casting

**Figure 1:**
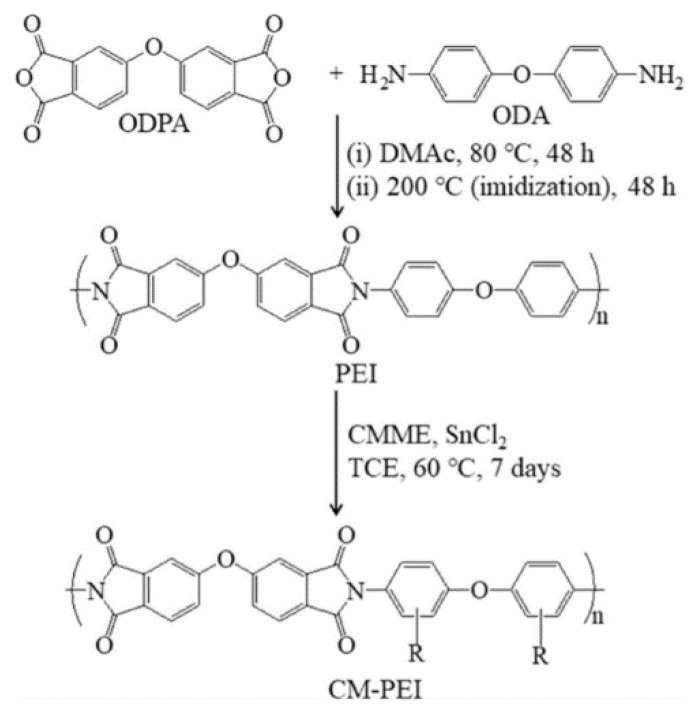
Synthesis and chemical structures of PEI and chloromethylated-PEI (CMPEI). Reprinted with permission from [34]. Copyright Elsevier, 2019.

Solution casting is the conventional method to fabricate AEM, which generally applies to soluble polymers, their blends, or copolymers [18]. In technical terms, this method comes with a four-step procedure, namely, the dissolution of polymer, introduction of the cation functional group via chloromethylation, polymer solution casting and quaternization. For instance, an alkalized poly(ether imide) (A-PEI) was synthesized by Oh et al. [34] in accordance to the steps given in Figure 1. The chemical structures of the A-PEI were confirmed by FTIR and NMR, which called the successful grafting of the ammonium group on the backbone of polymer. Based on the results obtained, the A-PEI membrane with a ratio between tin chloride and chloromethyl methyl ether (CMME) of 1:80 has shown the highest performance in IEC and hydroxide ionic conductivity at 1.23 meq g^−1^ and 44.2 mS cm^−1^, respectively. Parrondo et al. [35] demonstrated a novel AEM, synthesized by Friedel–Crafts acylation of 6-bromo-1-hexanoyl chloride on poly(phenylene oxide) (C6-PPO) followed by alkalization with trimethylamine (TMA). Interestingly, their results present neither a sign of improvement over the performance (IEC reported at 1.8 meq g^−1^ compared to membrane without quaternization of 1.7 meq g^−1^) nor its chemical stability in alkaline conditions, which is against their hypothesis regarding the potential of hexyl spacers of C6-PPO in stabilizing the AEM. Such an outcome is unusual because having spacer provides an advantage in side-chain flexibility, and subsequently enhances the anion conduction. This can be seen from the work of Zhu et al. [36], whereby an AEM was synthesized by incorporating rotatable ethylene oxide spacers into imidazolium-containing cationic side-chains. The PPO-based imidazolium AEM demonstrates a unique character whereby the facile rotation of C–O–C ether bonds contributes to the reduction in glass transition temperature and promotes the ion transportation. The hydroxide conductivity and IEC were reported at 45 mS cm^−1^ and 2.1 meq g^−1^, respectively, while the single cell AEMFC performance yielded a value of 437 mW cm^−2^ at 65 °C.

Nevertheless, the hydrophilic and rotatable ethylene oxide as spacer has high water affinity due to its tendency to form a hydrogen bond between ether groups and H_2_O. This hydrophilic property has proven to contribute to the water uptake of membrane, and thus may result in dimensional instability of AEMs at an elevated temperature. 

### 2.2. AEM Prepared via Solution Casting with Crosslinker

**Figure 2:**
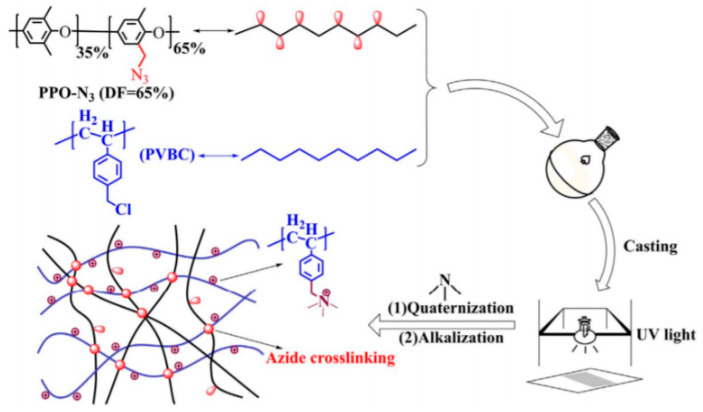
Crosslinking of PVBC-based AEMs with PPO-N_3_ by solution casting and UV irradiation methods. Reprinted with permission from [47]. Copyright Elsevier, 2017.

Covalent crosslinking is one of the effective techniques to solve the issue through: (1) bonding halomethyl groups with aromatic rings of neighboring polymer through a Friedel–Crafts reaction without external crosslinker, (2) bonding two functional groups on neighboring polymer lines together in the assistance of a micromolecular crosslinker, and (3) bonding two polymers together by the reaction between other groups and micromolecular crosslinker [25,42,43,44,45,46]. The aforementioned techniques on covalent crosslinking are not favorable due to their own limitations, which then leads to the proposal of a new route of covalent crosslinking by Lu et al. [46], using a macromolecule as crosslinker, and combines with the advantages of a semi-interpenetrated polymer network (semi-IPN) and crosslinking technique. The poly(vinyl acetal) (PVAc) was adopted as the macromolecular crosslinker to support the poly(vinylbenzyl chloride) (PVBC) through the interaction between quaternary ammonium (QA) groups and chloromethyl (–CH2Cl) groups. Although the hydrophilic nature of PVAc improved the conductivity performance and stability of the membrane at an elevated temperature, both excessive water uptake and swelling ratio will deteriorate the mechanical performance. Hence, a more hydrophobic polymer needs to be placed as the macromolecule crosslinker to suppress both water uptake and swelling ratio while forming the micro-phase separation of hydrophilic/hydrophobic polymers that assists in conducting the hydroxide. Xue et al. [47] have crosslinked PVBC with azidated poly(2,6-di-methyl-phenyleneoxide (PPO-N_3_) as shown in Figure 2. and resulted in 2–4 times higher tensile strength than those without crosslinked PVBC (15.8 MPa), yet with a comparable ion exchange capacity of 2.0 meq g^−1^ and an ionic conductivity of 3.5 mS cm^−1^. 

### 2.3. AEM Composite Membranes Incorporated with Inorganic Fillers

There are studies on the addition of inorganic components to the organic matrix of polymers such as titanium dioxide (TiO_2_), zirconium dioxide (ZrO_2_), aluminum oxide (Al_2_O_3_), bentonite, and graphene oxide (GO) to improve the morphological stability of the membrane [27,48,49,50,51,52]. The addition of inorganic components reduces the glass transition temperature and increases the amorphous phases of the polymer matrix, which increases its ionic conductivity [71]. For instance, Nonjola et al. [27] added TiO_2_ filler into the quaternary polysulfone membrane, which has shown a reduction in membrane swelling and improvement in morphological stability without compromising ionic conductivity. The atomic force microscopy (AFM) revealed that addition of TiO_2_ increases the surface roughness of the membrane that prevents fuel crossover and subsequently increases the current efficiency. In another study, Ion-Ebrasu et al. [53] introduced graphene oxide into Fumion® and found that the modified composite membrane with the loading of 0.50 *w*/*v*% graphene concentration increased the ionic conductivity to 113.27 mS cm^−1^ at 80 °C, outperforming the as-received Fumion® that was thermally stable up to 60 °C only. Movil et al. [33] have also shown that the incorporation of functionalized graphene oxide (FGO) into polyvinyl alcohol polydiallydimethylammonium chloride semi interpenetrating polymer networks (PVA/PDA/SIPNs) improve the OH^−^ conductivity and thermo-mechanical stability under high relative humidity. However, the addition of inorganic components in excess into the polymer matrix may produce high numbers of pores, leading to a fragile structure and fuel crossover, and subsequently hindering the performance of the membrane. 

### 2.4. AEM Prepared via Pore-Filling Copolymerization Technique

**Figure 3:**
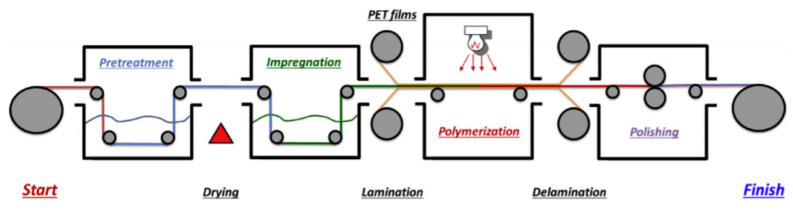
Schematic representation of the PAEM fabrication process using R2R equipment. Reprinted with permission from [72]. Copyright Elsevier, 2019.

The pore-filling membrane fabrication technique can be used to fabricate low swelling AEMs. In general, polymer electrolytes are impregnated into a porous substrate or monomers are impregnated into a porous substrate, followed by polymerization [72,73]. Next, the volatile solvents are removed by evaporation to form the pore-filling AEM. This method leverages the advantages of both polymer (flexible and high ionic conductivity) and porous substrate (improved mechanical strength). Kim et al. [54] have fabricated novel pore-filled AEMs with polytetrafluoroethylene (PTFE) and poly(*N*,*N*’-dimethylaminoethyl methacrylate-DVB) (poly(DMAEMA-DVB)) copolymer. It was found that the combination of both structurally stable ion-exchange sites (i.e., poly(DMAEMA-DVB)) and highly hydrophobic PTFE has effectively lessened the degradation of quaternary alkyl ammonium. In another study, Kim et al. [55] also showed that the impregnation of quaternary PPO (QPPO) in porous PTFE could improve the dimensional properties and ionic conductivity of AEM (from 12.7 mS cm^−1^ to 33.1 mS cm^−1^). Yang et al. [72] have upscaled the production of pore-filling AEM using a roll-to-roll (R2R) process, as shown in Figure 3**.** The AEM, which was produced from this process, has an IEC and water uptake of 1.645 meq g^−1^ and 34.47 %, respectively. These properties are comparable to the commercial Fujifilm AEM that has an IEC and water uptake of 1.840 meq g^−1^ and 56.58 %, respectively.

## 3. Design Consideration on Radiation-Grafted AEM 

The radiation-induced graft polymerization technique in fabricating AEMs is favorable because this technique is suitable for scale-up in industrial applications. Radiation has a strong penetrative power, which allows it to initiate the grafting process throughout the entire volume of polymer at relatively lower temperatures than the one with chemical initiators. The grafting penetration can also be adjusted accordingly to only functionalize the surface of polymers with the functional groups –COOH, –OR, –OH, –NH_2_, –SO_3_H, –R and their derivatives, modifying the surface properties without affecting the properties of bulk material [74]. In comparison to the conventional grafting method, radiation-induced graft polymerization promotes more controllable graft copolymers with a predetermined molecular weight and polydispersity of grafted chains. Moreover, the radiation-induced grafting method is simple, relatively clean and reprocessable. This technique is also capable of modifying the polymeric surface with a wide range of shapes and properties without requiring the presence of a catalyst or initiators. Particularly, the modified surface likely free from any impurities related to functional moiety [74]. 

Due to the versatility of this technique, there are a few key parameters, including the selection of radiation source, polymer backbone, monomer concentration, type of solvents and additives, dose rate, total dose, grafting atmosphere and temperatures, that will influence the properties of the final product of AEMs. One of the important and unique properties of radiation-grafted membrane is the degree of grafting (D.O.G) [62,75], which is used to determine the amount of functional group that is successfully grafted onto the base polymer. The D.O.G can be estimated from Equation (7), whereby the percentage of mass changes the membrane before being grafted (M_i_) and after being grafted (M_g_).

(7) $$D.O.G\;\left(\%\right)=\frac{M_{g}\left(g\right)-M_{i}\;\left(g\right)}{M_{i}\;\left(g\right)}\times 100\%$$

The higher the irradiation dose, the higher the D.O.G with more available sites for the cation head groups to attach with [64]; however, a high irradiation dose also causes damage to the base polymer and reduces mechanical strength [76]. A membrane with low ionic exchange capacity usually results in low ionic conductivity, while if the membrane has a high ionic exchange capacity, it is usually associated with high water uptake, which in turn increases the swelling ratio that contributes to the mechanical stresses that eventually damages the membrane structure integrity [69]. A balance between these properties is essential for commercial adoption.

### 3.1. Radiation Source 

The source of radiations can be from electromagnetic radiations such as gamma (γ)-ray, X-ray, ultraviolet (UV), or charged particles such as electrons and beta particles [13,62]. The highly penetrative characteristic of radiation also allows it to introduce uniform and controllable active sites for grafting initiation, depending on the radiation species, dose rate, irradiation dose, properties of base polymer and monomer, as well as grafting environment. In addition, there are fewer shape and structure restrictions on the polymer matrix for radiation grafting, which enables it to be applied on preformed polymers without any post-shaping issues [77]. The electron beam and gamma (γ)-ray are the two common sources used in radiation grafting. The electron beam is usually applied for the pre-irradiation method, where a high dose rate of electron beams is irradiated on the polymer backbone to produce free radicals. Meanwhile, gamma (γ)-ray is preferably applied in the simultaneous irradiation grating because it is the most penetrating of electromagnetic waves among the rest, but the grafting time is relatively longer than that of the electron beam [74]. However, gamma(γ)-ray offers much greater grafting efficiency by higher penetration [78]. Thus, the required grafting degrees are achievable at lower doses with gamma(γ)-ray in comparison to the electron beam. Furthermore, gamma(γ)-ray is more suitable for the preparation of grafted materials in large quantities for industrial enactment which relatively more economic, simpler, and robust. Irrespective of this, the selection of radiation source was still subjected to the targeted application and vital specification attached within the material functional scope. A membrane with a low ionic exchange capacity requires much lower grafting degrees merely on the surface of the polymeric materials. In these circumstances, the electron beam will be the most appropriate choice, as the penetration of electrons is considerably limited. While, contrastingly, gamma (γ)-ray fits well for the preparation of membrane with a high ionic exchange capacity for conferring higher grafting degrees.

### 3.2. Radiation Technique and Mechanism

**Figure 4:**
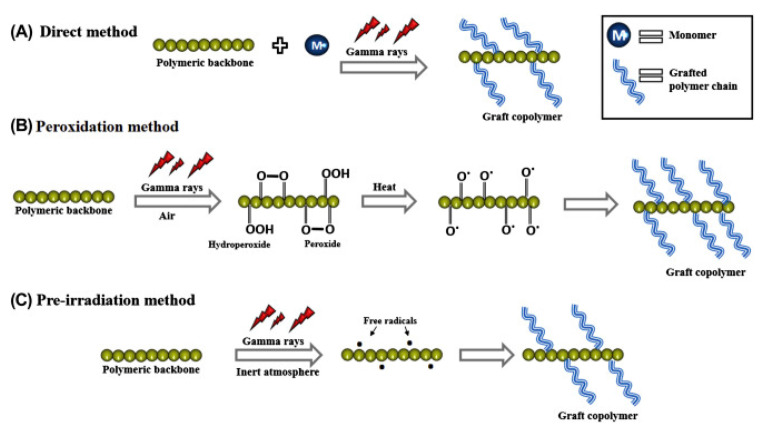
Schematic representation of the direct irradiation (**A**) and pre-irradiation methods (**B**) with the presence of oxygen, also known as the peroxidation method, and (**C**) without the presence of oxygen. Reprinted with permission from [79]. Copyright Elsevier, 2018.

There are two common radiation-induced polymer grafting methods: (i) pre-irradiation grafting, either in an inert atmosphere or vacuum, or in the presence of air or oxygen, and (ii) simultaneous irradiation, as shown in Figure 4. The selection between these techniques is subjected to the radiation source, reactivity of monomer and the polymer to be modified.

#### 3.2.1. Pre-Irradiation 

Pre-irradiation polymer backbone in an inert atmosphere or vacuum produces free radicals, which in turn act as a grafting site for the monomers. A high concentration of free radicals can be obtained with high-energy electron beams at a high dose rate, and grafting is performed instantaneously after the irradiation exposure [74]. In the presence of oxygen, the pre-irradiation of polymer backbone produces peroxides or hydroperoxides, which subsequently decompose into radicals at a higher temperature and initiate the grafting reaction upon interacting with monomers. The lifespan and stability of these radicals depend on the types of polymer backbone, as well as the storage conditions and durations, which permit the grafting process to be performed at a later time and away from the source of radiation [67,80]. The formation of homopolymer is in very minimal levels for this technique, as the activation of monomer by radiation is absent. Moreover, the degradation and crosslinking of the polymeric substrate are likely to take place during the course reaction, as the protection by monomer is missing as well. Besides, the efficiency of the grafting process is highly dependent on the radical lifetime of the polymer substrate [81]. Generally, the pre-irradiation method is suitable for production on a pilot scale.

#### 3.2.2. Simultaneous Irradiation Grafting 

In the simultaneous irradiation grafting method, both polymer backbone and monomers are irradiated simultaneously to produce free radicals. In principle, simultaneous irradiation is the simplest and most common grafting method to modify the polymer and is also appropriate for substrates sensitive to radiation. Undesirable effects of radiation degradation on the polymer matrix are greatly lessened by means of the protective effects from the monomers in the simultaneous irradiation grafting system. Furthermore, the greater number of radicals formed by irradiation on the polymer matrix in comparison to the one attained by the irradiation of monomers indicates an efficient grafting process. However, such a technique also leads to a higher chance of homo-polymerization and rapid deactivation of polymer backbone radicals due to recombination [82]. Simultaneous irradiation produces active sites in the polymer backbone, in the monomer and in the solvent, causing several unwanted side reactions that limit the degree of grafting and produce undesired homopolymers from the monomers. As such, this method requires inhibitors such as Fe^2+^ or Cu^2+^ or radical scavengers to suppress the formation of homopolymers. Hence, the choice of radiation technique depends on the characteristics of the polymer backbone and monomer and the scale of production.

### 3.3. Selection of Polymer Backbone, Irradiation Dose and Dose Rate 

There are two categories of polymer backbone used in radiation-induced graft polymerization: fluorinated (or partially fluorinated) polymers and nonfluorinated polymers. Fluorinated polymers such as ethylene tetrafluoroethylene (ETFE) and Nafion® have been extensively used as polymer backbones owing to their favorable chemical and thermal stability. For instance, Fang et al. [43] grafted VBC onto poly(ethylene-co-tetrafluoroethylene) (ETFE) using γ-ray in argon with a total irradiation dose of 90 kGy, and functionalized with DABCO, DCX and TMA at various degrees of grafting (D.O.G). The highest ionic conductivity achieved was 74 mS cm^−1^ at 80 °C. Ponce-González [83] studied the effect of the spacer groups on the alkaline stability of polymer. Butyl-spacer styrenic (labelled as C4-AEM) monomer and commercial VBC (labelled as C1-AEM) were grafted onto ETFE with an electron beam in air and aminated with MPY, respectively. They claimed that the IEC loss of C4-AEM after ageing in aqueous KOH (1 mol dm^−3^) at 80 °C for 28 days was 13%, approximately half of that of C1-AEM, which had an IEC loss of 27%. The degradation of C4-AEM was predominantly contributed to by the loss of Cl^−^ anions, but the N atoms were still retained on the polymer. Danks et al. [84] grafted VBC onto both polyvinylidene fluoride (PVDF) and fluorinated ethylene propylene (FEP), respectively. It was found that FEP-g-VBC membranes were thermally stable over a temperature range of 60 to 100 °C, with approximately an 18% drop in IEC at 60 °C over a period of 119 days and possessed good ionic conductivity at room temperature (reported at 0.01–0.02 S cm^−1^). Meanwhile, the PVDF-g-VBC membrane was unstable because the PVDF backbone degraded upon amination and alkaline treatment. Poynton et al. [70] have also grafted VBC onto ETFE membrane (ETFE-g-VBC) for AEMFC applications, and the membrane has demonstrated an IEC value of 1.24 meq g^−1^ with a considerably high water uptake of 155.4%. Despite the fact that high water uptake was reported, it may not necessarily be detrimental to the AEMFC performance, because water is of pivotal importance for ion conduction. The performance of ETFE-g-VBC was promising, with a peak power density of 240 mW cm^−2^ in comparison to 180 mW cm^−2^ of the benchmark MEA under identical conditions. The high water uptake of AEMs produced via radiation grafting was also confirmed by Horsfall et al. [85]. The reaction of radiation grafting promotes a high degree of grafting (D.O.G) on the membrane, which in turn facilitates a greater percentage of water uptake.

**Figure 5:**
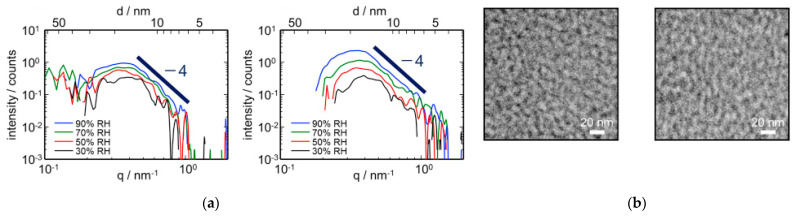
(**a**) Spherically shaped SAXS profile with a Porod’s slope of 4 and (**b**) its corresponding TEM image. Reprinted with permission from [89]. Copyright Elsevier, 2018.

Yoshimura et al. [65] have attempted to improve the alkaline stability of fluorinated polymer backbone by preventing the degradation of the imidazolium group caused by β-elimination and ring-opening hydrolysis reactions. They have grafted both hydrophobic styrene (St) and imidazolium containing methyl group (2-methyl-*N*-vinylimidazole (MNVIm)) onto ETFE (MNVIm/St-AEM) in argon atmosphere at room temperature, using a ^60^Co γ-ray source with a total irradiation dose of 80 kGy. The MNVIm/St-AEM was converted into terpolymergrafted membrane (MNVIm/En/St-AEM) upon treatment with 1 M KOH at 80 °C, where an ethene monomer (En) was introduced between St monomer and MNVIm monomer that is bonded to the ETFE. The ionic conductivity of MNVIm/En/St-AEM reduced drastically from 251 mS cm^−1^ (MNVIm/St-AEM) to 53 mS cm^−1^ but maintaining a similar swelling ratio of 23%. The alkaline stability of MNVIm/En/St-AEM was tested in 1 M KOH at 80 °C and its ionic conductivity was recorded. The addition of methyl protecting group (i.e., MNVIm) has shown enhancement in alkaline stability, with an ionic conductivity higher than 10 mS cm^−1^ at 800 h. To further understand the ionic conductivity channels of AEM in the presence of water [86], Yoshimura et al. [87,88] used the small-angle X-ray scattering technique (SAXS) to investigate the ionic conducting structure of AEMs grafted with 2-methyl-*N*-vinulimidazolium and styrene groups at different degrees of grafting under various relative humidity (RH) conditions. SAXS revealed that the mean distance between two ionic clusters was approximately 1.0 nm and the ionic conducting channels were disconnected at relative humidity (RH) < 80%. While in water, the ionic conductivity of these AEMs increased significantly because the ionic conducting channels were well connected. This structural model explained the reason why most power generation of AEMFC reported to date is low because the reduction reaction of water at the cathode will reduce the RH significantly, thus decreasing the ionic conductivity. Kimura et al. [89] used SAXS to investigate the morphology changes of quaternized poly(arylene ether) semi-block co-polymer in relation to RH at 40 °C. SAXS results showed that the peak intensity increased with humidity and a Porod’s slope of 4, as shown in Figure 5a, indicating that the periodic structure of hydrophilic domains in the AEM became more pronounced and the hydrophilic domains are in the form of a spherical shape, respectively. The SAXS shape analysis was consistent with the TEM results in Figure 5b.

**Figure 6:**
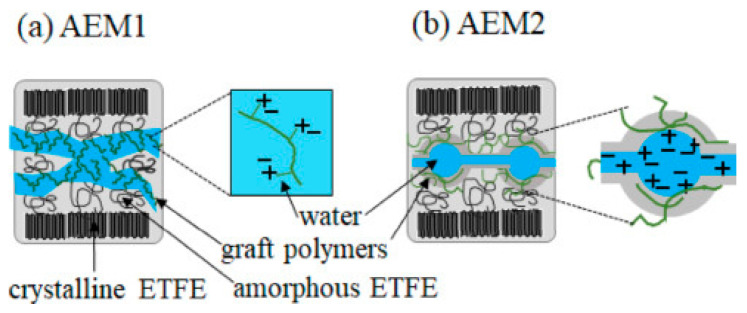
Schematic representation of ion channels of (**a**) parallel-grafted (AEM1) and (**b**) perpendicular-grafted (AEM2) ionic polymer into the ETFE polymer matrix. Reprinted with permission from [91]. Copyright Elsevier, 2018.

Building on the understanding of their previous work [65], Zhao et al. [90] have grafted imidazolium groups with St groups attached in perpendicular orientations onto ETFE (StIm-ETFE), with a total dose of 50 kGy at a dose rate of 10 kGy h^−1^, using γ-ray. This molecular design prevents the *β*-elimination reaction and hydrolysis of imidazolium ring, hence further improving the alkaline durability of ETFE. The grafting time was varied from 40 to 960 min to control the D.O.G. They found that StIm-ETFE with a D.O.G of 18% at 72 min exhibited an ion exchange capacity of 0.54 mmolg^−1^ and ionic conductivity of >50 mS cm^−1^ at a significantly low water uptake (~10%). The StIm-ETFE membrane showed no sign of degradation over 600 h in 1 M KOH solution at 80 °C, maintaining its ionic conductivity of 50–60 mS cm^−1^. They have also found that there was a critical ion exchange capacity (IEC_c_) point in the range of 0.7 to 0.8 mmol g^−1^, where above this IEC_c_ point, water uptake increased rapidly, contributed to by the switching of “reverse micelles” with water domains dispersed in the polymer matrix to ‘‘micelles’’ with graft polymer aggregates dispersed in the water matrix. Zhao et al. [91] also discovered that the ionic channels were microstructurally different depending on if imidazolium and styrene units were grafted parallelly (AEM1) or perpendicularly (AEM2) onto the ETFE membrane, respectively. The small-angle neutron scattering (SANS) with contrast variation method takes advantage of the inherent difference in scattering density properties between hydrogen and deuterium [92]. Upon hydration in heavy water (D_2_O), the hydrophilic ionic groups form ionic channels provide pathways to transport anion, while the hydrophobic ETFE polymer matrix maintains the membrane integrity by restricting the swelling. SANS revealed that both AEMs have a lamellar structure with alternate stacking between grafted polymer layers and crystalline ETFE layers, as shown in Figure 6. Nevertheless, the ionic channels of these AEMs were distinct from one another. AEM1 shows homogenous distributed ions in the ion channels, whereas AEM2 forms ionic clusters (caused by nanophase separation) with a size of ~2.2 nm, which improve the water diffusion and ionic conductivity of the membrane. 

Nonetheless, high irradiation doses may cause damage to the fluorinated polymer main chains, such as ETFE-based AEMs, leading to mechanical weakening [76]. As such, Wang et al. [70] have attempted to optimize the irradiation process by replacing propan-2-ol diluent with water, and further reduced the irradiation dose from 70 to 30 kGy, and VBC concentration from 20 vol% to 5 vol%. The results showed that ETFE-g-VBC with reduced irradiation dose preserved its high IEC of 2.01 mmol g^−1^ and improved mechanical properties with its Young’s modulus of 415 MPa, stress at break of 30% and elongation at break of 189%, which were higher than the one irradiated at 70 kGy. The peak power density of AEMFC with ETFE-g-VBC membrane was reported at 1.16 W cm^−2^ at 60 °C.

Non-fluorinated polymers, with a higher resistance to radiation damage, such as polyethylene (PE), low-density polyethylene (LDPE) and high-density polyethylene (HDPE)-based anion exchange membranes, were proposed as an alternative to EFTE. These non-fluorinated hydrocarbon-based polymers offer several advantages over the fluorinated polymer, such as lower costs, enhanced commercial precursor availability, and more possibilities for recycling the final products (no C-F content) [76]. LDPE-based AEM offers a cheaper alternative to other commercially available materials [64], with promising thermomechanical and chemical stability, as well as durability properties. Faraj et al. [93] studied UV-induced grafting of VBC onto LDPE and aminated with 1,4-diazabicyclo(2.2.2)octane (LDPE-g-VBC-DABCO) for electrolysis cell applications. Here, DABCO also functions as cationic crosslinker, which supports the formation of thin sheet anion exchange thermoplastic membrane. Although DABCO contains b-hydrogen atoms, DABCO is highly resistant to alkali degradation through Hofmann elimination because of its rigid cage structure [94]. The LDPE-g-VBC-DABCO membrane was examined in terms of its ionic conductivity, ion exchange capacity, water uptake, electrolytic cell tests, and hydrogen permeability. They found that the electrochemical performance of LDPE-g-VBC-DABCO was comparable with the benchmark commercial membrane at that time, and the electrolysis cell with this membrane was able to produce approximately 30 cc/min of hydrogen at 20 bar, over more than 500 h. It showed that the hydrogen permeation rate of LDPE-g-VBC-DABCO, which was a safety concern in electrolysis applications, was in the range of 10^−13^ mol cm^−1^ s^−1^ kPa^−1^. However, the LDPE-g-VBC-DABCO membrane displayed instability due to its low resistance in an alkaline environment. Wang et al. [76] compared the performance of radiation-grafted ETFE-based AEM and LDPE-based AEM. The LDPE-based AEM was able to withstand an irradiation dose of up to 100 kGy without showing mechanical failure, while only an irradiation dose of 30 kGy was used in ETFE-based AEM. It was found that that the Cl^−^ conductivity of radiation-grafted LDPE-based AEM (76 mS cm^−1^ at 80 °C) was slightly higher than that of ETFE-based AEM (60 mS cm^−1^ at 80 °C). However, the elasticity of LDPE-AEM after aging in alkaline at 80 °C improved drastically, with the elongation at break of LDPE-based AEM at least an order larger than that of ETFE-based AEM. Similarly, Meek et al. [95] have studied the alkaline stability of radiation-grafted LDPE-based AEM at temperatures above 60 °C. The LDPE-based AEMs were subjected to a 4.5 MeV electron beam irradiation at a dose of 100 kGy in air and subsequently grafted with vinylbenzyl chloride (VBC) and aminated with trimethylamine (TMA), *N*-methylpyrolidine (MPY) and *N*-methylpiperidine (MPIP) head groups, respectively. They found that TMA-type LDPE-based AEM has the highest Cl^−^ ionic conductivity (100 mS cm^−1^ at 80 °C) and lowest alkaline degradation compared to the other two types. LDPE-g-VBC-TMA fabricated via the simultaneous irradiation technique using γ-ray in N_2_ saturated solution at a dose rate of 2 kGy h^−1^ and a total radiation dose of 20 kGy by Gupta et al. [69,96] also showed a similar ionic conductivity of 90 mS cm^−1^ at 50 °C. 

While LDPE-based AEM has superior ionic conductivity and fast water transport kinetics, it is still prone to environmental stress cracking, particularly in the long-term operation of fuel cells and electrolysis cells. Limited by the membrane thickness for a fuel cell and electrolysis cell, Wang et al. [63] have proposed to replace the LDPE-based AEM with high-density polyethylene-based AEM (HDPE-based AEM) to enhance the mechanical properties of AEM. They have produced HDPE-based AEM using a 4.5 MeV dynamic continuous electron beam at a high dose rate electron beam of 100 kGy in air. The HDPE-based AEM was then grafted with VBC saturated in N_2_ for 4 h at 50 °C. This HPDE RG-AEM showed an enhancement in tensile properties (52% higher than that of LDPE), fuel cell performance (voltage degradation up to 7%) and operational stability (400 h of continues operation), yet with a comparable ionic conductivity (~214 mS cm^−1^), ionic exchange capacity (~2.44 mmol g^−1^) and water uptake (~155%) properties to LDPE-based AEM, which is contributed to by the changes in nanomorphology that improved the water transport mechanism after radiation grafting. Apart from HPDE, ultra-high molecular weight polyethylene (UHMWPE) is another class of low-cost and extremely tough polyethylene that can be used as the base polymer. Sherazi et al. [67] grafted VBC onto UHMWPE powder with γ-ray, which is subsequently compressed into thin AEM membrane and quaternized with TMA (UHMWPE-g-VBC-TMA). The highest ionic conductivity obtained in this investigation was 47.5 mS cm^−1^ at 90 °C, which was much lower than that of LDPE-based and HDPE-based AEMs discussed above. However, its ionic exchange capacity was notably constant (~0.58 meq mol g^−1^), even after 120 h of submersion in 10 M NaOH at 60 °C. Later, Sherazi et al. [68] quaternized UHMWPE-g-VBC with modified guanidine, 1,1,3,3-tetramethyl-2-n-butylguanidine (UHMWPE-g-VBC-TMBG). The IEC values increased with the total irradiation dose, with the highest IEC obtained for this membrane being 0.56 meq g^−1^ with a total irradiation dose of 25 kGy. This trend agreed with other studies. However, they also observed that the maximum ionic conductivity was achieved at 27.7 mS cm^−1^ at 90 °C with a total irradiation dose of 5 kGy. Beyond this point of irradiation dose, the ionic conductivity of the membrane decreased, which may be attributed to the excessive phase separation that disrupts the connectivity of ion conduction channels, resulting in higher resistivity. 

Espiritu et al. [64] grafted VBC onto various sources of polyethylene, consisting of casting membranes of both low-density polyethylene (cast-LDPE) and linear low-density polyethylene (cast-LLDPE) from pellets supplied by Sigma Aldrich, and commercial membrane films from British Polyethene Industri plc (BPI-LDPE), VMR International (VMR-LDPE), ENTEK microporous (ENTEK-UHMWPE), using γ-ray with a dose rate of 2 kGy h^−1^, and functionalized with TMA. They reported that an increase in total irradiation dose from 10 to 20 kGy resulted in an increase in D.O.G. of all the polyethylene membrane because more active sites were formed for VBC monomers to graft onto the polyethylene backbone. The BPI-LDPE-g-VBC membrane showed the highest value of IEC (3.2 meq g^−1^) and D.O.G (74.6%) among the listed polyethylene membranes, respectively. The membrane also showed an extremely stable ionic conductivity of 0.11 S cm^−1^ in a durability test over a period of 7 months under nitrogen at 80 °C. The AEMFC based on the BPI-LDPE-g-VBC membrane has demonstrated a peak power density of 608 mW cm^−2^ at 50 °C, with low fuel crossover. In another study, Espiritu et al. [75] investigated the effect of irradiation dose rate on the degradation of radiation-grafted VBC onto polyethylene-based (LDPE and HDPE) and EFTE-based AEMs and functionalized with TMA, respectively. The polyethylene-based membranes were both prepare via simultaneous irradiation with γ-ray with dose rate ranges from 30 to 2000 kGy h^−1^, while ETFE-based membrane was pre-irradiated with electron beam at a dose rate of 400 kGy h^−1^. Interestingly, they found that AEMs, regardless of fluorinated or non-fluorinated polymer backbone, that were treated with a high irradiation dose rate (above 400 kGy h^−1^) resulted in a lower loss of IEC (less than 15%) in percentage over a period of 2 months compared to those treated at a low irradiation dose rate (up to 50% loss of IEC). They concluded that polymer subjected to high irradiation dose rate exhausts available oxygen rapidly and prevents any dissolved oxygen from penetrating into the polymer that degrades the polymer through oxidation. Table 2 summarizes and compares the performance of polyethylene-based and ETFE-based AEMs at different irradiation doses and dose rates. 

In 1996, Shell commercialized a type of aliphatic polyketone (PK), which is a terpolymer of carbon monoxide, ethylene and propylene. This relatively new type of polymer has excellent thermomechanical properties, high chemical stability and high resistance to hydrolysis. Nevertheless, its unique chemical resistance property also contributes to its difficulty to graft chemically. Kim et al. [97] are among the first group that used γ-irradiation to graft VBC onto PK membrane and aminated with TMA (PK-g-VBC-TMA). They have successfully achieved a D.O.G of 98% with a VBC monomer concentration of 70 wt% and at the total irradiation dose of 70 kGy. They found that the performance of PK-g-VBC-TMA outperformed a Nafion 117, which has an ionic conductivity of 78 mS cm^−1^, an IEC of 0.8 meq g^−1^, water uptake of 24% and swelling ratio of 21%. Both ionic conductivity and the IEC of PK-g-VBC-TMA were recorded as 310 mS cm^−1^ and 1.2 meq g^−1^, respectively, which was supported by its maximum water uptake of 35%. Although its water uptake was much higher than that of Nafion, the swelling ratio of PK-g-VBC-TMA was comparable to that of Nafion, which indicated that the mechanical strength of PK-g-VBC-TMA has a high potential for commercial applications. 

Recently, Samiego et al. [13] selected the biodegradable cellulose acetate (CA)-based polymer as the backbone for the first time because it is low-cost and environmentally benign. The use of natural polymers as a base for AEM in fuel cell and electrolysis cell applications offers a green alternative to modern energy production. The CA polymer was grafted with VBC and functionalized with TMA, using γ-ray at an irradiation dose of 40 kGy. An average ionic conductivity of up to 163 mS cm^−1^ at 40 °C was achieved; however, the performance of the CA-g-VBC-TMA membrane deteriorated drastically beyond 60 °C due to excessive water uptake. By reducing the total irradiation dose treatment to 25 kGy, CA-g-VB-TMA membrane was able to operate in up to 70 °C, with a maximum ionic conductivity of 93 mS cm^−1^_._ Apart from LDPE and HPDE, ultra-high molecular weight polyethylene (UHMWPE) is another class of low-cost and extremely tough polyethylene that can be used as the base polymer. 

## 4. Conclusions

The development of AEM for AEMFC and AEMEC applications is still in its infancy stage. Conventional membrane fabrication methods such as solution casting, covalent crosslinking, filler inclusion and pore-filling have been used to produce AEMs. Compared to the conventional methods, the radiation-induced grafting technique has the advantages of simplicity, cost saving, energy efficiency and being green. In addition, this advanced membrane-processing technology is highly customizable, where the irradiation parameters such as radiation sources, rate, dose and environment can be adjusted to control the initiation and propagation velocity of the grafting process at the desired modification depth. Radiation-induced grafting enables the control of hydrophilic and hydrophobic domains to ensure good micro/nanophase separation in the AEM to facilitate high ionic conduction. Radiation grafting spacers onto base polymers also improves the chemical stability properties of AEMs. With the booming of the hydrogen economy and increasing demand of low-cost green hydrogen production, both AEMFC and AEMEC have gained substantial attention because of their potential to reduce the cost of power and hydrogen production, respectively. Although the durability of AEMs has remarkably improved over the years, the durability of AEMs remains the main challenge and requires further improvement to achieve commercial readiness.

## Figures and Tables

**Table 1:** Properties of anion exchange membrane (AEM) with different preparation methods.

Modification Method	Polymer	Reagent(s) for Cationic Head Group	Water Uptake (%)	Swelling Ratio (%)	Tensile Strength (MPa)	IEC (meq g^−1^)	Ionic Conductivity (mS cm^−1^)	Cell Performance (mW cm^−2^)	Ref.
Simple solution casting without modification	PEI	Trimethylamine (TMA)	40.3	19.2	21.5	1.23	44.2 (at 90 °C)	-	[34]
	C6-PPO	TMA	-	-	-	1.80	1.2 (at 25 °C)	-	[35]
	PPO	1,2-bis(2-(2-methylimidazole)ethoxy)ethane	150.0 (at 60 °C)	18.0	-	2.10	45.0 (at 60 °C)	437.0 (at 65 °C)	[36]
	Poly(fluorenyl ether ketone sulfone) (PFEKS)	(i) TMA (ii) 1-methylimidazole (IM)	(i) 59.0 (ii) 48.0	-	-	(i) 1.80 (ii) 1.60	(i) 22.3 (ii) 17.1	-	[37]
	PPO	1-benzyl-3-methyl-4-butyl-1,2,3-triazolium iodide	128.0	35.0	-	1.21	61.6	-	[38]
	PTFE	Quaternized 1,4-diazabicyclo[2.2.2]-octane (DABCO)	24.0	17.0	32.0	-	51.0 (at 55 °C)	146 (at 50 °C)	[39]
	Polysulfone	DABCO	122.7	12.3	24.0	1.68	0.9	-	[40]
	CPPO/BPPO	TMA	137.4	-	28.0	2.10	27.6	-	[41]
Covalent crosslinking	Polysulfone	Quaternary phosphonium	100.0	15.0	-	1.23	38.0 (at 20 °C)	-	[25]
	ETFE	TMA	11.7	0.8	55.8	1.07	15.3 (at 60 °C)	-	[42]
	Ethylene-co-tetrafluoroethylene (ETFE)	TMA	64.4	-	-	2.11	73.5 (at 80 °C)	48.0 (at 40 °C)	[43]
	Polysulfone	N-methyl-pyrrolidinone (NMP)	22.7	-	-	1.33	-	-	[44]
	Poly(acrylene ether sulfone) (PSF)	TMA	50.0	24.0	-	0.73	5.5	-	[45]
	PVBC	PVAc	139.1	26.3	14.2	1.26	29.0	124.7 (at 40 °C)	[46]
	PVBC	PPO-N_3_	19.8	6.9	59.5	1.95	14.8 (at 20 °C)	11.0 (at 60 °C)	[47]
Composite membrane with inorganic fillers	Polysulfone Filler: TiO_2_	TMA	39.0	-	-	-	125.2 (at 21 °C)	-	[27]
	Polysulfone Filler: ZrO_2_	Triethyl amine (TEA)	18.3	-	28.4	0.90	14.6	250.0 (at 60 °C)	[48]
	Poly(vinyl alcohol) (PVA) Filler: Al_2_O_3_	KOH	-	-	-	-	0.6	-	[49]
	PVA Filler: Bentonite	KOH	65.0	-	-	-	110.0	-	[50]
	Chitosan/PVA Filler: GO	NaOH	138.4	-	48.6	0.37	0.1	-	[51]
	Polystyrene Filler: GO	Sodium dodecyl benzene sulfonate (SDBS)	-	-	-	>1.80	-	-	[52]
	Fumion® Filler: GO	NMP	18.4	-	2890.6	3.16	113.2 (at 80 °C)	-	[53]
Pore-filling	PTFE filled with poly(DMAEMA-DVB)	p-xylene dichloride (XBC)	19.9	-	44.7	1.40	128.0	7.7 (at 60 °C)	[54]
	PTFE filled with QPPO	TEA	14.6	18.3	275.0	1.44	33.1	-	[55]
	Poly(styrene) filled with VBC-DVB	TMA	25.8	10.1	125.8	2.04	0.4	-	[56]
	Polyethylene filled with VBC-DVB	Pyridine	70.0	4.0	-	0.95	-	-	[57]
Conventional copolymerization	Hyper-branched PVBC-grafted-VBC	TMA	38.6	36.3	6.77	1.26	50.8 (at 30 °C)	-	[58]
	Polychloromethylstyrene-*b*-polycyclooctene-*b*-polychloromethylstyrene (PCMS-*b*-PCOE-*b* -PCMS)	TMA	64.7	13.6	184.5	1.83	179.0 (at 80 °C)	-	[59]
	Quaternized poly(arylene ether sulfone)	DABCO	74.1 (at 90 °C)	29.6 (at 90 °C)	-	1.86	51.8 (at 90 °C)	64.0 (at 60 °C)	[60]
	Quaternized chitosan-polyacrylamide/polystyrene (QCS-PAM/PS)	(2,3-epoxypropyl)trimethylammonium chloride (EPTMAC)	64.5	19.0	43.9	0.93	6.0 (at 80 °C)	-	[61]
Radiation grafting copolymerization	Low density polyethylene-grafted-VBC (LDPE-g-VBC)	TMA	-	-	-	2.53	85.0 (at 60 °C)	-	[62]
	Cellulose acetate-g-VBC	TMA	176.0	45.9	-	1.41	93.0 (at 70 °C)	-	[13]
	HDPE-g-VBC	TMA	155.0	21.0	35.0	2.44	214.0 (at 80 °C)	2550.0 (at 80 °C)	[63]
	LDPE-g-VBC	TMA	285.0	55.6	11.2	3.20	120.0 (at 70 °C)	607.8 (at 50 °C)	[64]
	MNVIm/En/St-AEM	2-methylimidazolium	128.0	23.0	-	1.05	55.0 (at 80 °C)	-	[65]
	ETFE-g-VBC	TMA	155.4	-	-	1.24	-	240.0 (at 50 °C)	[66]
	Polyethylene-grafted-VBC (PE-g-VBC)	TMA	13.7	14.6	-	0.49	47.5 (at 90 °C)	-	[67]
	PE-g-VBC	1,1,3,3-tetramethyl-2-n-butylguanidine (TMBG)	4.5	7.3	-	0.33	27.7 (at 90 °C)	-	[68]
	LDPE-g-VBC	TMA	-	-	-	2.30	90.0 (at 50 °C)	180 mV	[69]
	ETFE-g-VBC	TMA	40.0	-	18.2	-	34.0 (at 50 °C)	2.8 (at 50 °C)	[10]
	ETFE-g-VBC	TMA	57.0	32.0	27.0	2.13	68.0 (at 80 °C)	-	[70]

**Table 2:** Properties of anion exchange membrane (AEM) at a different irradiation dose and dose rate.

AEM	Irradiation Dose/Dose Rate (kGy/Gy h^−1^)	D.O.G (%)	Water Uptake (%)	Tensile Strength (MPa)	IEC (meq g^−1^)	Ionic Conductivity (mS cm^−1^)	Cell Performance (mW cm^−2^)	Ref.
StIm-ETFE	Dose: 50 Grafting time: 40 to 72 min	8 to 18	5 to 10	-	0.26 to 0.54	17 to 50 (at 60 °C)	-	[90]
BPI-LDPE-g-VBC	Dose: 10 to 20	50.4 to 74.6	285	-	2.4 to 3.2	90 to 110 (at 60 °C)	608 (at 50 °C)	[64]
ETFE-g-VBC	Dose: 40 to 30	89 to 76	57 to 53	262 to 416	2.13 to 2.01	68 to 60 (at 80 °C)	1160 (at 60 °C)	[70]
LDPE-based AEM	Dose: 50 to 100	102 to 143	97 to 104	275	2.63 to 2.87	64 to 76	1450 (at 80 °C)	[76]
UHMWPE-g-VBC-TMBG	Dose: 3 to 5	8.5 to 12.5	3.25 to 4.5	-	0.22 to 0.33	14.4 to 27.7 (at 90 °C)	-	[68]
LDPE-g-VBC-TMA	Dose rate: 67 to 2000	68 to 65	-	-	2.8 to 2.7	84 to 99 (at 60 °C)	-	[75]
HDPE-g-VBC-TMA	Dose rate: 35 to 67	58 to 66	-	-	2.6 to 2.9	84 to 101 (at 60 °C)	-	[75]

## Data Availability

Not applicable.
